# Differential Patterns of Microbiota Recovery in Symbiotic and Aposymbiotic Corals following Antibiotic Disturbance

**DOI:** 10.1128/mSystems.01086-20

**Published:** 2021-04-13

**Authors:** Shavonna M. Bent, Carolyn A. Miller, Koty H. Sharp, Colleen M. Hansel, Amy Apprill

**Affiliations:** a Woods Hole Oceanographic Institution, Woods Hole, Massachusetts, USA; b Johnson State College, Johnson, Vermont, USA; c Roger Williams University, Bristol, Rhode Island, USA; University of Technology Sydney

**Keywords:** *Astrangia poculata*, SSU rRNA gene, microbiome, extracellular superoxide

## Abstract

Microbial relationships are critical to coral health, and changes in microbiomes are often exhibited following environmental disturbance. However, the dynamics of coral-microbial composition and external factors that govern coral microbiome assembly and response to disturbance remain largely uncharacterized. Here, we investigated how antibiotic-induced disturbance affects the coral mucus microbiota in the facultatively symbiotic temperate coral Astrangia poculata, which occurs naturally with high (symbiotic) or low (aposymbiotic) densities of the endosymbiotic dinoflagellate Breviolum psygmophilum. We also explored how differences in the mucus microbiome of natural and disturbed *A. poculata* colonies affected levels of extracellular superoxide, a reactive oxygen species thought to have both beneficial and detrimental effects on coral health. Using a bacterial and archaeal small-subunit (SSU) rRNA gene sequencing approach, we found that antibiotic exposure significantly altered the composition of the mucus microbiota but that it did not influence superoxide levels, suggesting that superoxide production in *A. poculata* is not influenced by the mucus microbiota. In antibiotic-treated *A. poculata* exposed to ambient seawater, mucus microbiota recovered to its initial state within 2 weeks following exposure, and six bacterial taxa played a prominent role in this reassembly. Microbial composition among symbiotic colonies was more similar throughout the 2-week recovery period than that among aposymbiotic colonies, whose microbiota exhibited significantly more interindividual variability after antibiotic treatment and during recovery. This work suggests that the *A. poculata* mucus microbiome can rapidly reestablish itself and that the presence of *B. psygmophilum*, perhaps by supplying nutrients, photosynthate, or other signaling molecules, exerts influence on this process.

**IMPORTANCE** Corals are animals whose health is often maintained by symbiotic microalgae and other microorganisms, yet they are highly susceptible to environmental-related disturbances. Here, we used a known disruptor, antibiotics, to understand how the coral mucus microbial community reassembles itself following disturbance. We show that the *Astrangia poculata* microbiome can recover from this disturbance and that individuals with algal symbionts reestablish their microbiomes in a more consistent manner compared to corals lacking symbionts. This work is important because it suggests that this coral may be able to recover its mucus microbiome following disturbance, it identifies specific microbes that may be important to reassembly, and it demonstrates that algal symbionts may play a previously undocumented role in microbial recovery and resilience to environmental change.

## INTRODUCTION

Most corals maintain beneficial relationships with microalgae in the family Symbiodiniaceae (1), which support their ability to deposit aragonite skeletons and form reefs (2). Environmental disturbances, such as elevated seawater temperatures and excessive UV radiation, cause disruption of this critical symbiosis between Symbiodiniaceae and corals. Prolonged disruption of this symbiotic relationship can often lead to coral death, although recolonization by the algae is possible (3), sometimes by more tolerant algae (4, 5). Research over the last 2 decades has uncovered additional relationships that corals harbor with other protists, bacteria, archaea, fungi, and viruses (6–9), clearly designating corals as multipartner symbioses. Thermal bleaching and disruption of the coral-Symbiodiniaceae relationship are often correlated with altered coral microbiome community composition, which is hypothesized to be due to the altered physiological state of the coral (10–12). While these thermal tolerance experiments are useful to understand the dynamics of coral microbiomes in the face of a variety of environmental disturbances, we still have yet to identify specific drivers of bacterial and archaeal assembly and stability in healthy hosts.

Microbial assembly within a host is still one of the most understudied aspects of animal-microbial relationships, including those of corals. For coral larvae, microorganisms are typically obtained from the surrounding seawater (13, 14), although some corals that brood (release developed larvae instead of eggs) vertically transmit microbes to their young (15). Antibiotic-exposure experiments in adult corals have yielded some insight into microbial assembly dynamics. After disturbance with antibiotics, Acropora muricata was slowly recolonized with microbes that are generally found in the surrounding seawater, but their community structure differed from that of the typical coral microbiome, suggesting that the colonizing microbes were opportunistic and able to colonize only in the absence of coral-associated microbes (16). Over the course of 4 days, the coral’s bacterial community (from coral mucus, tissue, and skeleton together) began to regain a structure similar to that of their pretreatment state, suggesting some coordinated control over the microbiome reassembly (16). In analyses of the surface mucus layer alone, different dynamics were observed. After antibiotic treatment, Porites astreoides colonies harbored elevated abundances of microorganisms, including potential pathogens (*Verrucomicrobiaceae* and *Vibrionaceae*), in their mucus compared to those of nontreated corals, and this mucus microbiota resembled communities identified in aged coral mucus (17). Additionally, upon return to the reef, the antibiotic-treated colonies experienced increased rates of necrosis and mortality compared to those of control colonies (17), suggesting that the surface mucus acts as a selective and dynamic medium for microbial selection and that the loss of protective microorganisms contributed to the mortality (17). Indeed, because there are continuous disturbances facing corals today (18, 19) and there is strong evidence that coral-associated microorganisms provide resilience to the holobiont (20, 21), there is a need to identify patterns of coral microbiome reassembly following disturbance and to better understand the factors that govern microbial organization in coral mucus.

An emerging hypothesis is that extracellular production of reactive oxygen species (ROS) by coral and/or associated microorganisms may play a beneficial role in the physiology and health of the coral host (22, 23), as previously observed for other eukaryotes (24, 25). In particular, extracellular production of the ROS superoxide (O_2_^●−^) is involved in numerous basal physiological processes, including cell signaling, cell differentiation, wound repair, and growth in micro- and macroorganisms spanning bacteria to animals (26–28). Although elevated levels of intracellular ROS in coral cells have been implicated in oxidative stress and bleaching (the expulsion of algal symbionts from the host), extracellular ROS production has been measured in healthy corals, coral-associated heterotrophic bacteria, and Symbiodiniaceae (22, 29–33). Due to the multiple production and degradation processes and sources, deciphering the biological source of extracellular superoxide production in corals is challenging, and simplified experimental systems examining microbial colonization could shed light on the importance of bacterial-derived superoxide production.

The northern star coral, Astrangia poculata, is an emerging system for studying multipartner symbioses because it possesses unique traits not found in most tropical coral species. *A. poculata* is a stony coral that naturally occurs as brown colonies, harboring high densities of the Symbiodiniaceae Breviolum psygmophilum (symbiotic colonies), and white colonies, with densities of *B. psygmophilum* near detection limits (aposymbiotic colonies) (1, 34). Colonies of both symbiotic states coexist in the same environments, and both types associate with similar, predictable communities of bacteria and archaea (35). Thus, *A. poculata* offers a unique opportunity to study coral-microbial relationships in colonies with a range of algal symbiont densities without requiring laboratory-induced thermal stress/bleaching.

In this study, we investigated how antibiotic-induced disturbance alters the *A. poculata* mucus microbiome. Specifically, we followed the succession of the *A. poculata* mucus microbiome in symbiotic and aposymbiotic colonies over the course of 2 weeks after antibiotic treatment and compared these results to those of control treatments (non-antibiotic exposure). Our results indicate that while antibiotic exposure significantly altered the composition of the mucus microbiome, the *A. poculata* mucus microbiota in all treated corals recovered to a state comparable to that of the control following 2 weeks of exposure to ambient seawater. Furthermore, symbiotic colony microbiomes shared a more consistent reassembly pattern than did aposymbiotic colonies. We identified six bacterial taxa that played a prominent role in reestablishing the *A. poculata* mucus microbiome, suggestive of their importance for structuring the microbiome. We also tracked the extracellular superoxide levels at the surface of symbiotic and aposymbiotic colonies before and after disturbance to monitor the role of mucus-derived superoxide in total superoxide production. We found that the antibiotic exposure and subsequent microbial community changes did not influence the net production of extracellular superoxide by the coral holobiont.

## RESULTS

### Experimental overview.

**FIG 1:**
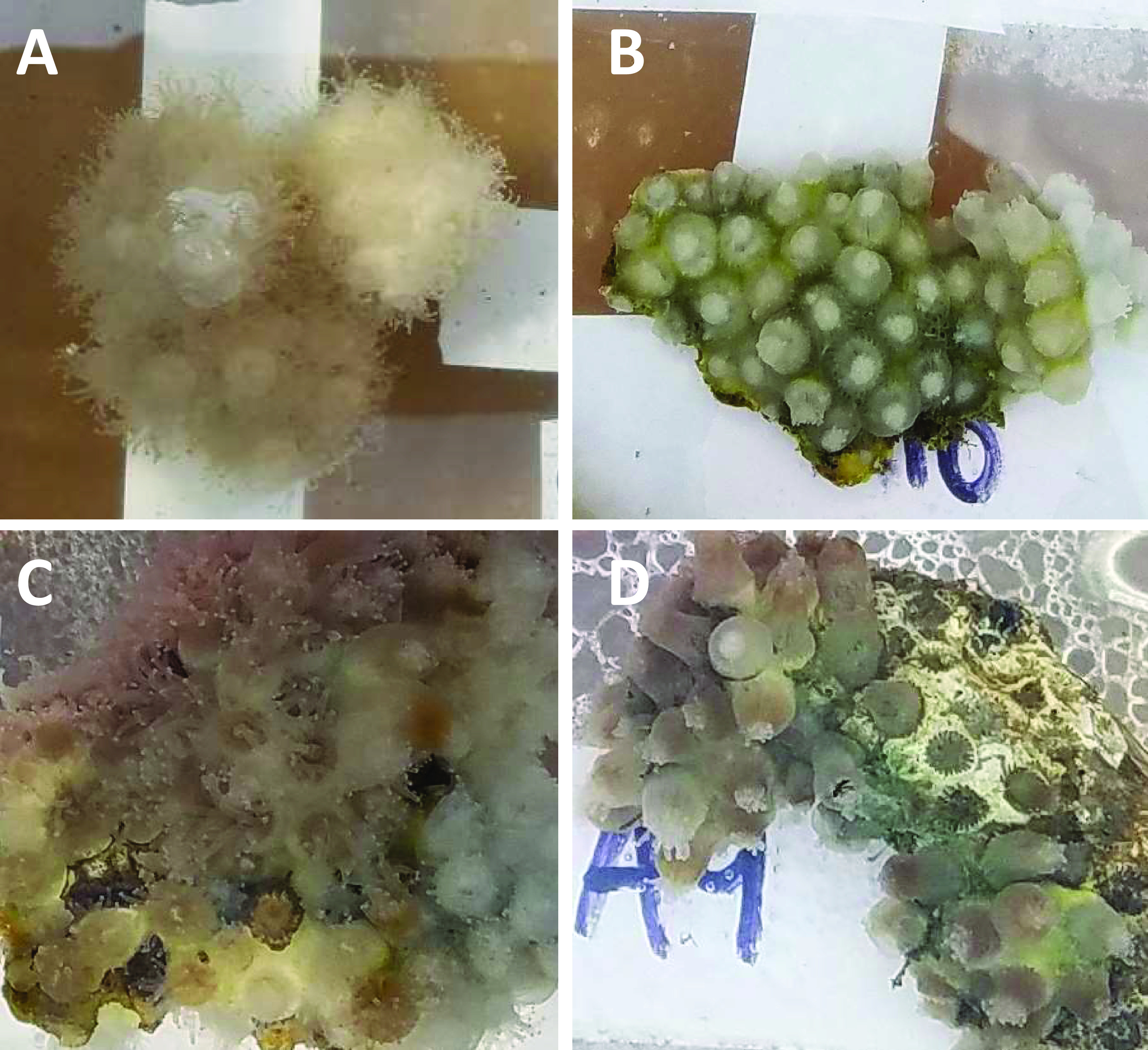
*Astrangia poculata* colonies during the course of treatment. Antibiotic-treated colonies (B and D) demonstrated phenotypic changes, such as swollen and contracted polyps. These changes were observed during treatment and up to 3 days after treatment was completed, while control colonies (A and C) appeared generally the same throughout the experiment. Symbiotic state did not appear to relate to the occurrence of these physical changes.

Symbiotic and aposymbiotic *Astrangia poculata* colonies were divided equally into control and antibiotic treatment groups (*n* = 4 or 5 per symbiotic state, per treatment; see Table 2). The antibiotic-treated corals were exposed to an antibiotic cocktail (Table 1) in filter-sterilized natural seawater, with doses administered daily over the week. The control corals were maintained in filter-sterilized seawater. In the antibiotic treatments, up to 3 days following exposure, both symbiotic and aposymbiotic colonies exhibited visible phenotypic changes, including swollen polyps with retracted tentacles (Fig. 1B and D), while control colonies did not exhibit such changes (Fig. 1A and C). Mucus and seawater were sampled for analysis of associated bacterial and archaeal communities by sequencing the V4 region of the small-subunit rRNA (SSU rRNA) gene, with mucus collected from all colonies immediately before antibiotic treatment (pretreatment), immediately following antibiotic treatment (0 h), and 96 h, 1 week, and 2 weeks after treatment (Table 2). Extracellular superoxide concentrations at colony surfaces were also measured, during the pretreatment time point and immediately after antibiotic or control treatments (0 h), and were not further examined in the experiment because measurements were consistently below detection limits. Extracellular superoxide production was also measured in axenic cultures of bacteria isolated from *A. poculata* mucus.

**TABLE 1:** Description of antibiotics used to treat coral colonies

Antibiotic	Amt in cocktail (100 ml)	Spectrum of efficacy	Antibiotic target
Streptomycin	2,043 mg	Gram-negative and Gram-positive bacteria	Protein synthesis inhibitor
Kanamycin	943 mg	Gram-negative bacteria	Protein synthesis inhibitor
Ampicillin	100 mg	Gram-positive bacteria	Cell wall inhibitor
Erythromycin	12 mg	Gram-positive bacteria (some fungi)	Protein synthesis inhibitor
Nystatin	8 mg	Fungi	Fungal cell membrane inhibitor
Gentamycin	5 mg	Gram-negative bacteria	Protein synthesis inhibitor

**TABLE 2:** Number of coral colony mucus or seawater samples collected over the course of the experiment for microbiome analysis

Sample type		Pretreatment	0 hours after	96 hours after	1 wk after	2 wks after
Control treatment	Symbiotic *A. poculata*	5	4	4	4	3
	Aposymbiotic *A. poculata*	5	4	4	3	4
	Seawater	1^*a*^	2	2	2	1

Antibiotic treatment	Symbiotic *A. poculata*	5	4	3	4	4
	Aposymbiotic *A. poculata*	5	3	3	4	4
	Seawater	1^*a*^	2	1	2	2

a The same sample was utilized for both pretreatment time points.

### Antibiotics disrupt microbial community structure, with recovery after 2 weeks.

**FIG 2:**
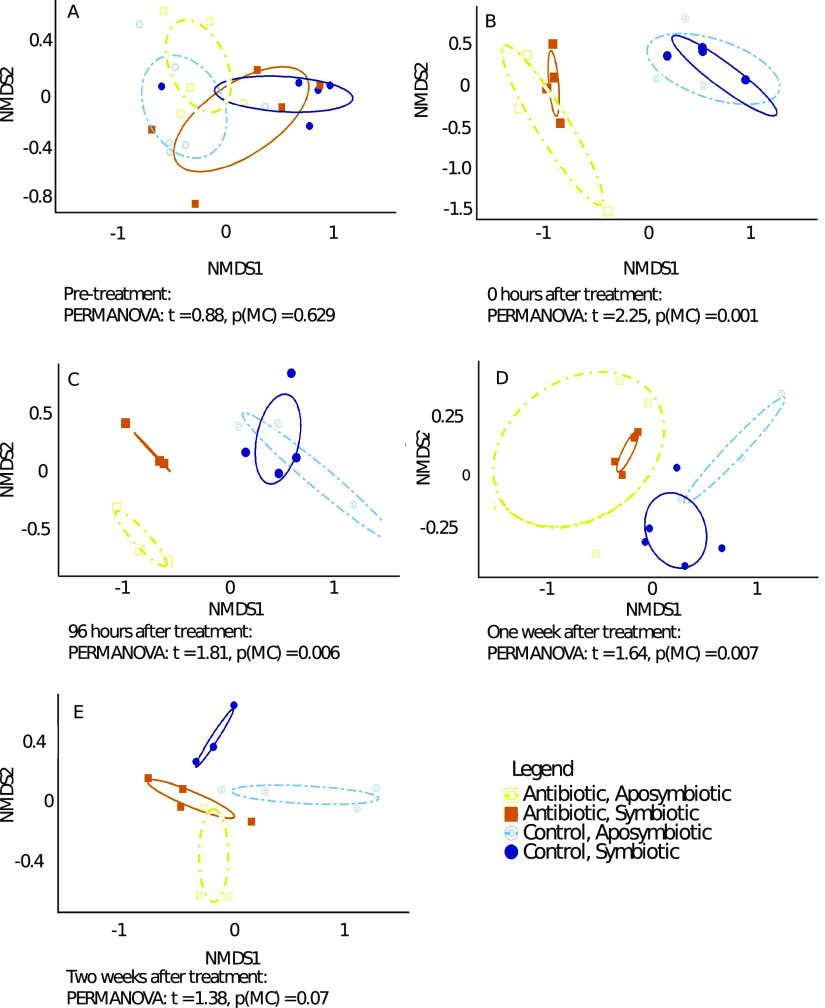
Nonmetric multidimensional scaling (NMDS) analysis of *A. poculata* mucus microbial communities for pretreatment (A), 0 h after treatment (B), 96 h after treatment (C), 1 week after treatment (D), and 2 weeks after treatment (E). Communities are grouped by treatment (color of line) and by symbiotic state (dashed versus solid line type). The PERMANOVA results listed at the bottom of each NMDS plots are comparisons between the control and treatment colonies for each time point (regardless of symbiotic state).

Sequencing of bacterial and archaeal SSU rRNA genes resulted in 4,000 to 274,392 sequences per sample, which were classified into 97% similarity operational taxonomic units (OTUs), resulting in 11,169 OTUs in the data set. The structure of the *A. poculata* mucus microbiome was compared using permutational multivariate analysis of variance (PERMANOVA) with a Monte Carlo (MC) simulation to confirm significance. At the pretreatment time point, the control and treatment microbiomes were not significantly different {Fig. 2A; PERMANOVA: *t* = 0.88, Monte Carlo *P* value [*P*(MC)] = 0.629}, but significant differences were observed between treatment and control microbiomes at 0 h, 96 h, and 1 week after treatment, regardless of symbiotic state (Fig. 2B to D) [PERMANOVA: *t* values from 2.25 to 1.64, *P*(MC) values from 0.001 to 0.007; see Table S1 in the supplemental material]. At 2 weeks, there was no significant difference between the treatment and control colonies [PERMANOVA: *t* = 1.38, *P*(MC) = 0.07; Fig. 2E].

10.1128/mSystems.01086-20.2 TABLE S1 List of significance tests and results, including analysis of similarity (ANOSIM) and permutational analysis of variance (PERMANOVA). Download Table S1, PDF file, 0.6 MB. Copyright © 2021 Bent et al. 2021 Bent et al. https://creativecommons.org/licenses/by/4.0/ This content is distributed under the terms of the Creative Commons Attribution 4.0 International license.

Throughout the experiment, the microbial community structure of the control colonies shifted slowly over time, resulting in a significantly different community at the end of the experiment compared to the pretreatment community (Table S1). However, between each adjacent sampling time point, no significant differences were observed (Table S1). Further, seawater community composition was consistent between treatment and control groups at each time point (Table S1), suggesting that all colonies received similar access to seawater microorganisms.

**FIG 3:**
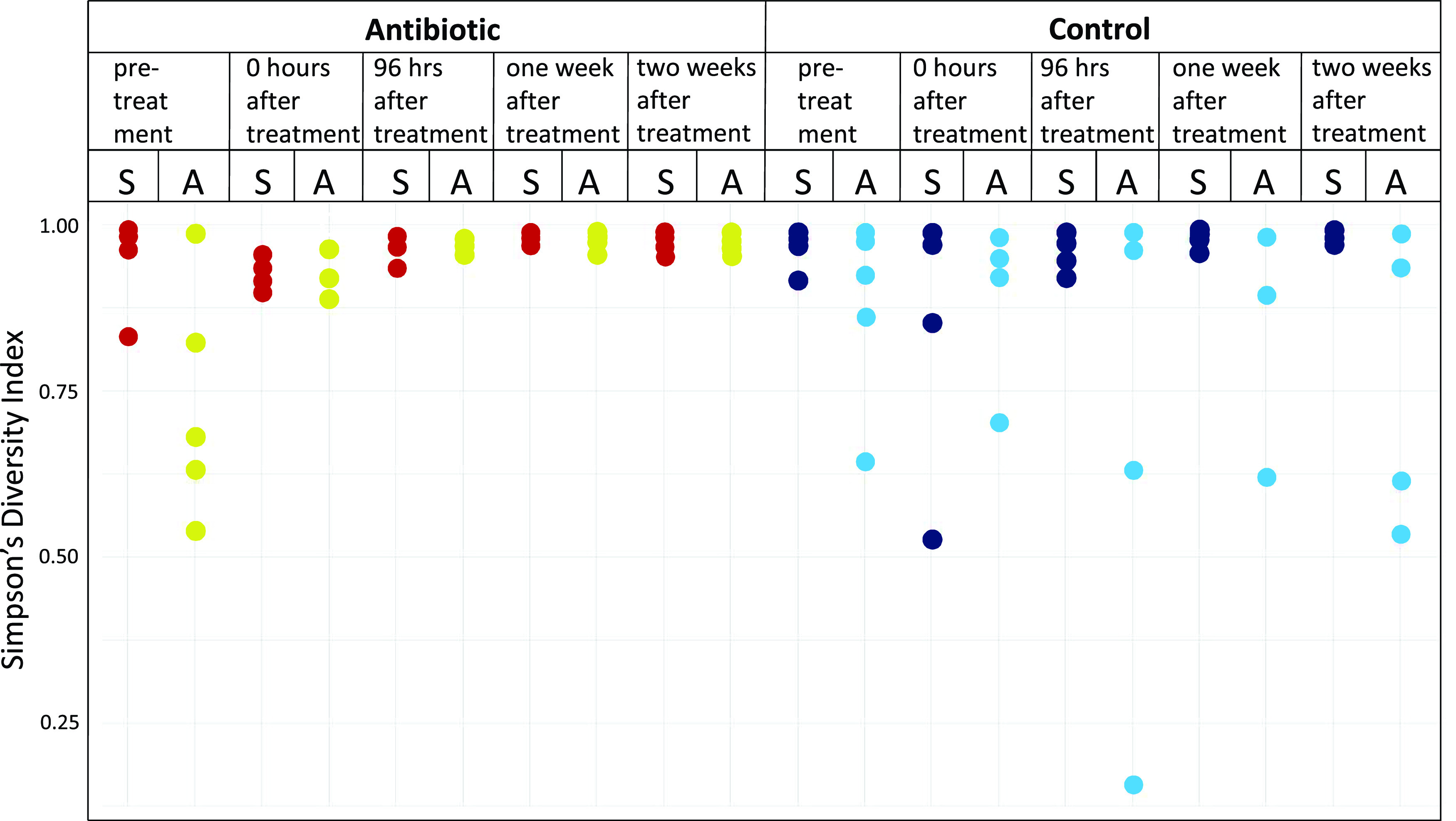
Alpha diversity of the *A. poculata* mucus microbiome, calculated using Simpson’s diversity index.

While beta diversity varied greatly between the *A. poculata* treatment groups, alpha diversity remained similar between treatments. Some individual colonies displayed lower levels of Simpson’s diversity index at specific treatment times; none of these differences were significant [PERMANOVA, *P*(MC) > 0.05; Fig. 3].

### Symbiotic colonies demonstrate high microbiome similarity during disturbance and recovery.

**FIG 4:**
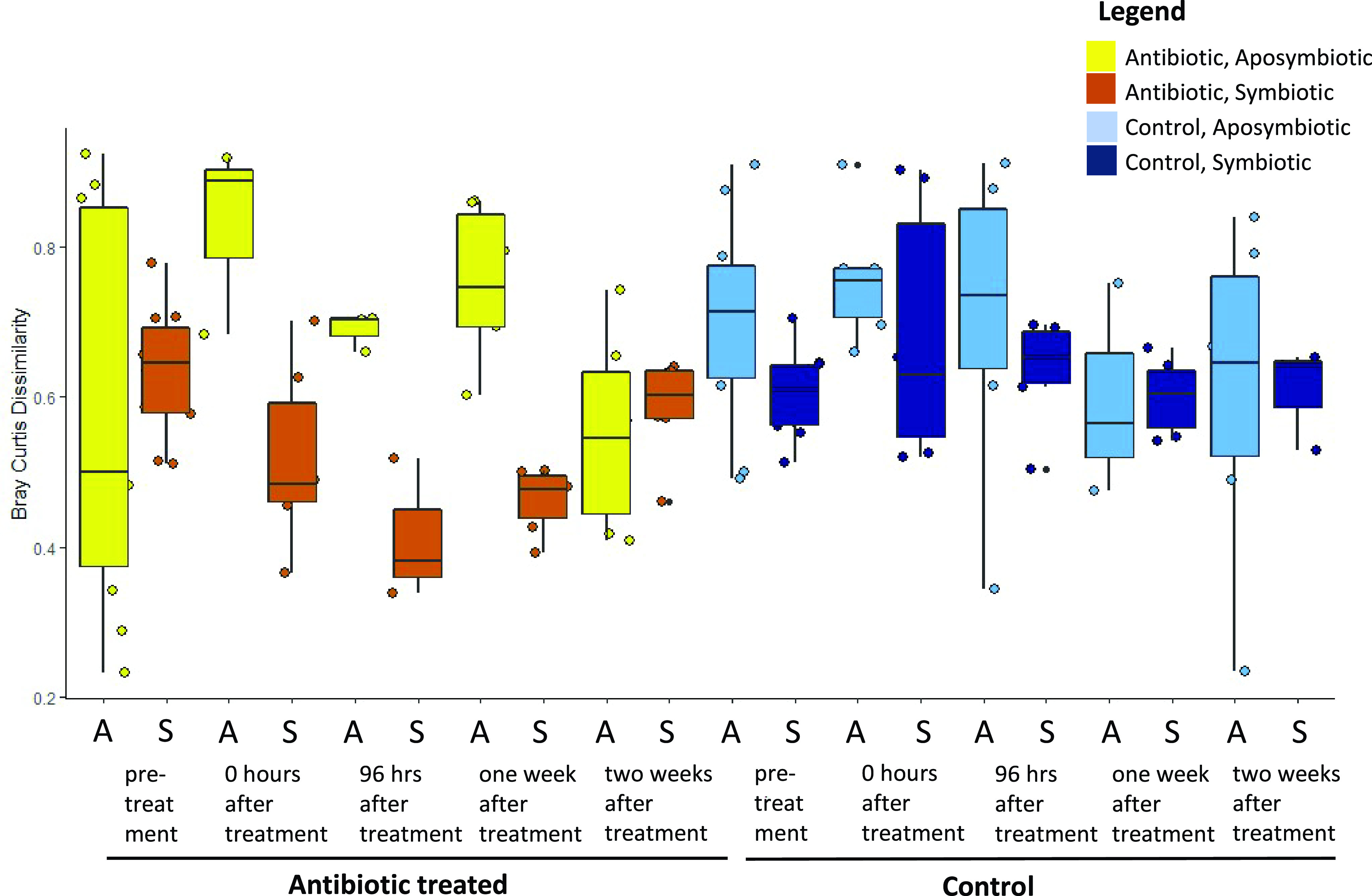
Average Bray-Curtis dissimilarity value by treatment over time, grouped by symbiotic state. After treatment with antibiotics, symbiotic colonies had lower dissimilarity values during the first week of recovery compared to those of the aposymbiotic colonies, indicating more within-group similarity in symbiotic colonies. After 2 weeks of recovery the symbiotic states were similar to each other and the control colonies in terms of average dissimilarity. The differences between symbiotic states are significantly different (three-way ANOVA using treatment, time, and symbiotic state; *P* < 0.05).

Ellipses in the beta-diversity analysis (Fig. 2) of the SSU rRNA gene data designate 95% covariance of mucus microbiomes between groups, and as indicated by the areas of 95% ellipses around the symbiotic and the aposymbiotic colonies, symbiotic colonies maintained higher within-group similarity than did aposymbiotic colonies. To more quantitatively examine this trend, we examined the average Bray-Curtis dissimilarity of symbiotic and aposymbiotic colonies in each treatment group over time (Fig. 4). Aposymbiotic colonies had significantly higher intercolony Bray-Curtis dissimilarity levels compared to those of the symbiotic colonies (three-way analysis of variance [ANOVA] using treatment, time, and symbiotic state; *P* < 0.05). At 2 weeks posttreatment, the differences in dissimilarity were less pronounced.

### Specific bacteria are sensitive or resistant to antibiotics, and others recolonize following disturbance.

**FIG 5:**
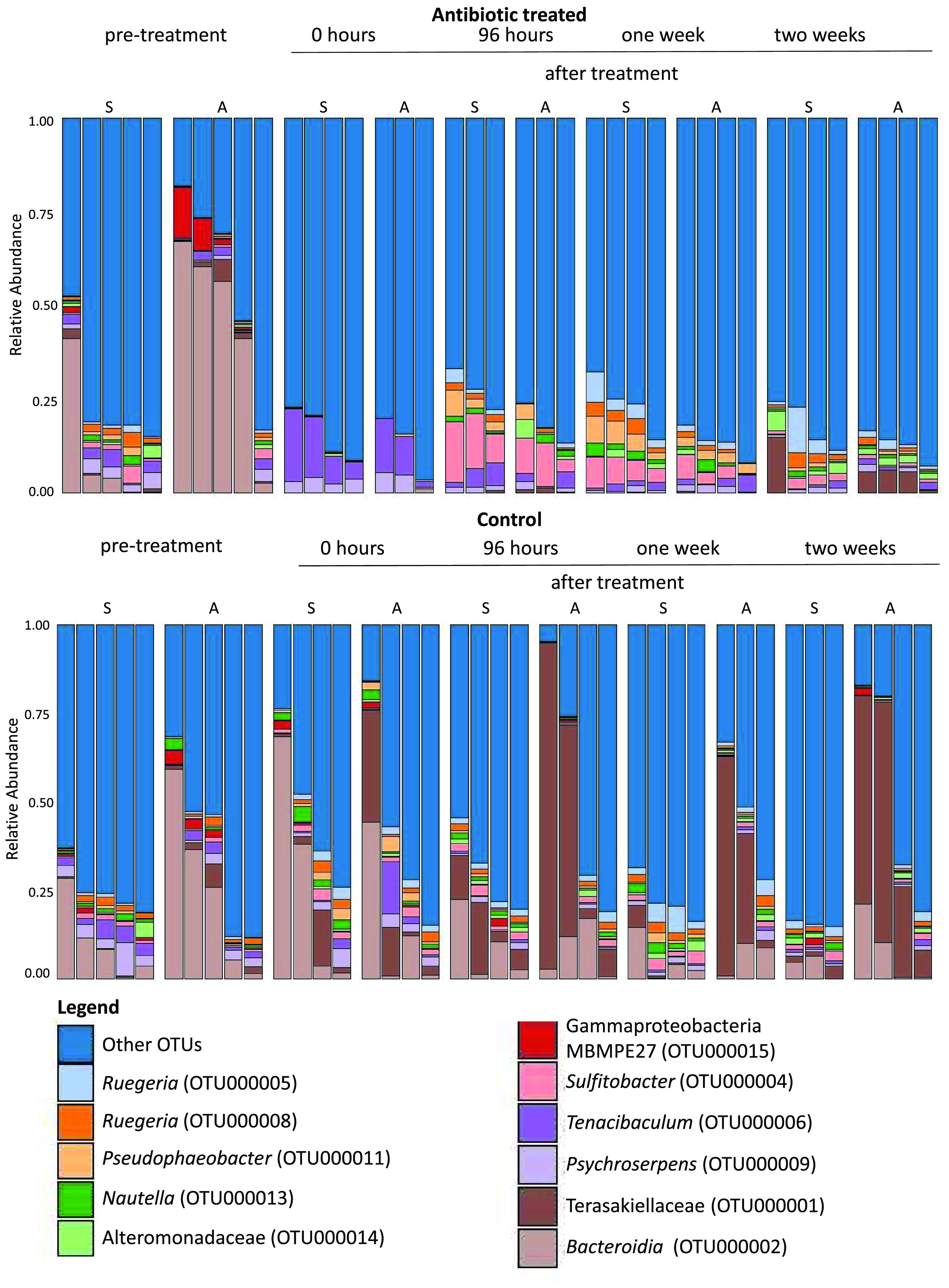
Relative abundances of the 11 OTUs responsible for the dissimilarity between treatment groups, determined using similarity and percentages routine (SIMPER) analysis.

**FIG 6:**
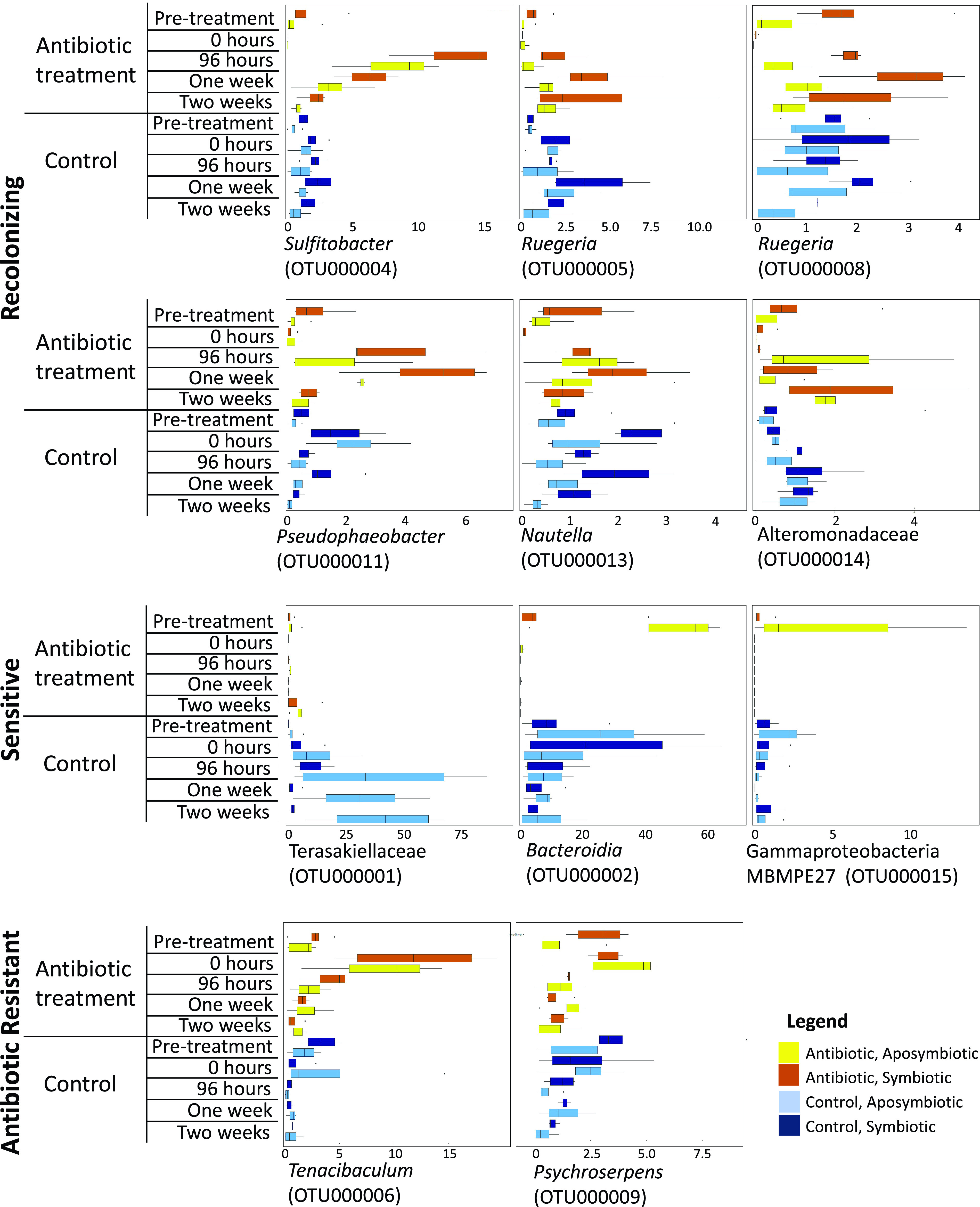
Relative abundances of the 11 OTUs responsible for the dissimilarity between treatment groups, grouped according to their recolonizing, sensitive, or resistant dynamics displayed during the experiment. The horizontal axis is relative abundance (%) of the microbiome.

Similarity and percentages routine (SIMPER) was used to investigate OTUs responsible for dissimilarity between treatment groups over time. We identified 11 OTUs, all affiliated with bacteria, responsible for these differences. In pretreatment corals, these OTUs comprised 15 to 75% of the microbiome, suggesting that the SIMPER analysis accounted for fluctuations within existing members of the *A. poculata* mucus microbiome (Fig. 5). We grouped the 11 OTUs identified as important to dissimilarity into three main categories: (i) resistant bacteria, including OTUs whose relative abundance did not decrease with antibiotic exposure, (ii) sensitive bacteria, or OTUs that decreased in relative abundance compared to that of controls following antibiotic exposure and did not recover during the experiment, and (iii) recolonizing bacteria, or OTUs which initially decreased in relative abundance following antibiotic disturbance but recovered to abundances near that of the control treatment after 2 weeks or less (Fig. 6). We hypothesize that these taxa recolonize the coral, because they are detected in the seawater. The OTUs identified as sensitive to antibiotics included Terasakiellaceae (OTU000001), *Bacteroidia* (OTU000002), and gammaproteobacteria MBMPE27 (OTU000015) (Fig. 6). These bacteria were present in mucus from the control colonies throughout the experiment but were observed in the antibiotic colonies only prior to treatment. Two OTUs, *Tenacibaculum* (OTU000006) and *Psychroserpens* (OTU000009), were identified as resistant to antibiotics, meaning they were present before and after antibiotic exposure (Fig. 6). The recolonizing group was comprised of six OTUs that were entirely eliminated, or nearly completely removed, from the community immediately following treatment (Fig. 6). However, in the days or weeks following treatment, their presence was reestablished. Generally, after disappearing, the abundance of members of this group spiked up to levels comparable to or higher than their pretreatment values. Over the course of the 2-week recovery period, the relative abundance slowly decreased again back to levels on par with the pretreatment values. This group was comprised of *Sulfitobacter* (OTU000004), *Ruegeria* (OTU000005 and OTU000008), *Pseudophaeobacter* (OTU000011), *Nautella* (OTU000013), and Alteromonadaceae (OTU000014).

### Net production of extracellular superoxide by isolated bacteria but not *A. poculata* colonies.

Extracellular superoxide concentrations at the surface of *A. poculata* colonies were measured at each colony during pretreatment and immediately after antibiotic exposure, for both symbiotic and nonsymbiotic colonies. In all colonies and treatments, extracellular superoxide levels were below the limit of detection. Since we are measuring steady-state superoxide concentrations, which are a function of opposing production and decay rates, these results indicate that there is no net production of superoxide stemming from the colonies. Further, there was similarly undetectable superoxide at the surface of coral colonies following antibiotic exposure.

**FIG 7:**
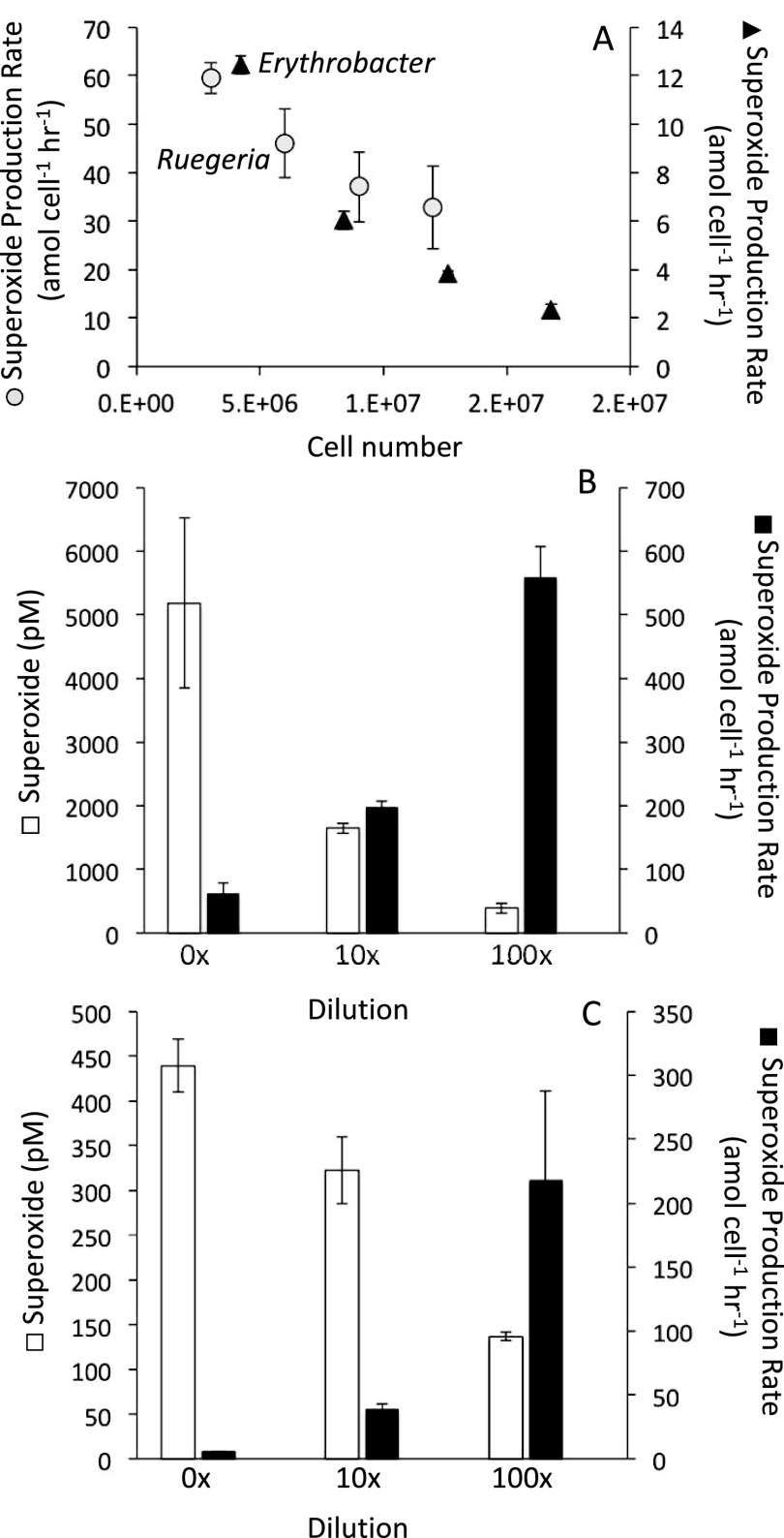
Extracellular superoxide production by mucus-associated Ruegeria meonggei and Erythrobacter vulgaris cultures. (A) Cell-normalized superoxide production rates as a function of cell number measured in cell loading experiments (*n* = 4). Steady-state superoxide concentrations and cell-normalized superoxide production rates for (B) *R. meonngei* and (C) *E. vulgaris* measured in dilution experiments.

Because many coral-associated bacterial species produce extracellular superoxide (22), we examined extracellular superoxide in two bacterial strains that were previously isolated from the mucus of *A. poculata* collected from the same location as our experimental corals. These are closely related to two common members of the *A. poculata* mucus microbiome (35), Ruegeria meonggei and Erythrobacter vulgaris. Extracellular superoxide was produced by both species during growth in nutrient-rich media (e.g., when isolated from the host). Superoxide production varied as a function of cell density, ranging from 2.34 amol O_2_^●−^ cell^−1 ^h^−1^ (cell loading experiments) to 217.9 amol O_2_^●−^ cell^−1 ^h^−1^ (dilution experiments) in *E. vulgaris* (Fig. 7A). Cell-normalized production rates varied more drastically for *R. meonggei*, with rates spanning 32.75 amol O_2_^●−^ cell^−1 ^h^−1^ (cell loading experiments) to 558.3 amol O_2_^●−^ cell^−1 ^h^−1^ (dilution experiments) depending on cell density (Fig. 7A). During the cell loading experiments, aliquots from the same culture were progressively added to the filter measuring superoxide production. While the overall levels of superoxide increased for *R. meonggei* and decreased slightly for *E. vulgaris*, the per cell production decreased drastically, demonstrating an immediate response to increased cell number. In contrast, for dilution experiments, mid-exponential-grown cultures were 10× and 100× diluted and analyzed for superoxide production after acclimation for 4 h (Fig. 7B and C). As observed for the cell loading experiments, while superoxide concentrations were higher in the presence of more cells, the cell-normalized superoxide production rates were considerably lower.

## DISCUSSION

In this study, microbial communities associated with the surface mucus layer of the coral *A. poculata* significantly changed after antibiotic disturbance. Following a 2-week period of exposure to natural seawater microorganisms, the surface mucus microbiome of antibiotics-exposed *A. poculata* colonies resembled that of untreated control *A. poculata* colonies. Throughout this period the microbial community structure associated with the aposymbiotic colonies exhibited significantly more variability among the individual colonies than that associated with the symbiotic colonies. Eleven bacterial OTUs were primarily responsible for the changes in community structure, including two antibiotic-resistant OTUs, three antibiotic-sensitive OTUs that did not recolonize the mucus, and six antibiotic-sensitive OTUs that likely recolonized *A. poculata* mucus. This study also determined that while bacteria isolated from *A. poculata* produce extracellular superoxide in culture, there is no detectable net superoxide production by the *A. poculata* holobiont, regardless of symbiotic state or the composition of the mucus microbiome.

Antibiotic exposure resulted in a phenotypic change to the symbiotic and aposymbiotic corals, observed as swollen polyps. The swollen polyps were only observed under antibiotic exposure, suggesting that this change is influenced by alterations in the microbiome of the coral. Swollen polyps have also been observed in *A. poculata* during winter or in cooler seawater temperature conditions in the field. This phenotypic change is related to quiescence, a metabolically dormant period during which *Astrangia poculata* ceases feeding (35). Similar to the shift seen in winter months in wild *A. poculata* colonies (35), the initial mucus microbiome shift in both symbiotic states may be a consequence of decreased food uptake by the host. Our corals were sampled during the beginning of summer, and thus the swollen polyp morphology is not likely due to low temperatures (temperature was maintained at levels above the winter threshold throughout the experiment and was consistent between control and antibiotic-treated tanks; see Table S2 in the supplemental material). Winter quiescent corals do not respond to tactile stimulation (36), but tactile response was not specifically evaluated during this experiment and is worth examining in future experimental manipulations. The microbiota of winter-related quiescent colonies shifts significantly, suggesting that animal metabolic state, and perhaps host feeding, is correlated with microbiome makeup (35). Antibiotic exposure has been documented to cause phenotypic responses in some animals, such as changes in weight and metabolism-related hormone levels (37, 38); thus, it is not surprising that *A. poculata* also exhibits a visible phenotypic response to antibiotic treatment.

10.1128/mSystems.01086-20.3 TABLE S2 Temperature and light data collected using a HOBO data logger. Readings were taken every 5 minutes throughout the course of the experiment. Download Table S2, EPS file, 0.5 MB. Copyright © 2021 Bent et al. 2021 Bent et al. https://creativecommons.org/licenses/by/4.0/ This content is distributed under the terms of the Creative Commons Attribution 4.0 International license.

Antibiotic exposure resulted in significant changes to the coral mucus microbiome. We identified 11 OTUs that had distinct patterns during the recovery of the colonies, 9 of which demonstrated a decrease in relative abundance in antibiotic treatments relative to that of pretreatment and control colonies. Three of these “sensitive microbes,” associated with Terasakiellaceae, *Bacteroidia*, and the Gammaproteobacteria MBMPE27 group, were not detectable in any of the later time points in the mucus microbiome of the treatment animals. Because these are not common bacteria in seawater, they may be specific to the *A. poculata* mucus and require transfer from other *A. poculata* colonies or other hosts for recolonization. Consistent with this idea, Terasakiellaceae are commonly associated with the cold-water coral *Paragorgia arborea* (39). Future experiments in which disturbed *A. poculata* colonies are exposed to untreated *A. poculata* colonies are needed to address the hypothesis that disturbed colonies can recruit microbes from other neighboring, healthy colonies. The six OTUs that were sensitive to antibiotics but reemerged in greater numbers in the mucus after 2 weeks were all generally common bacteria within the seawater (Fig. S1) used during this experiment (*Sulfitobacter*, two OTUs of *Ruegeria*, *Pseudophaeobacter*, *Nautella*, and Alteromonadaceae). This observation suggests that recruitment/recolonization from an environmental reservoir of bacteria may be related to the recovery of the *A. poculata* surface microbiome.

10.1128/mSystems.01086-20.1 FIG S1 Relative abundance (%) of 11 selected OTUs in seawater samples. See Fig. 6 for further information on the groupings of recolonizing (yellow), sensitive (green), and antibiotic-resistant (red) OTUs and their identifications. Download FIG S1, PDF file, 0.03 MB. Copyright © 2021 Bent et al. 2021 Bent et al. https://creativecommons.org/licenses/by/4.0/ This content is distributed under the terms of the Creative Commons Attribution 4.0 International license.

Bacterial OTUs affiliated with *Tenacibaculum* and *Psychroserpens* appeared to be resistant to the antibiotic cocktail in this experiment. *Tenacibaculum* increased in relative abundance directly following treatment, which may reflect the decreasing abundances of other microbiota rather than growth of *Tenacibaculum.* The relative abundance of *Psychroserpens* increased slightly following treatment with antibiotics, and abundances slowly decreased over the 2 weeks of recovery. *Tenacibaculum* has been recovered from the surface of stony and soft corals, including black band diseased colonies (40, 41), and *Psychroserpens* dominated the microbiomes of fish with amoebic gill disease (42). While *Tenacibaculum* and *Psychroserpens* bacteria may not be the causative agent of these diseases, their ability to flourish under disease conditions, as well as disruption of the normal microbiome, suggests that they may be opportunistic in nature.

An unexpected outcome of this experiment was the differential patterns of microbiome recovery observed in the symbiotic and aposymbiotic corals following antibiotic disturbance. Symbiotic and aposymbiotic colonies showed similar phenotypic responses (swollen polyps, retracted tentacles) to the antibiotic treatment. Immediately following antibiotic exposure and throughout the 1-week recovery period, the microbiome composition among the antibiotic-treated symbiotic colonies was more consistently similar than that among the antibiotic-treated aposymbiotic colonies (Fig. 4). Together, these findings suggest that symbiotic *A. poculata* colonies exert a stronger influence on recovery of the microbiome than do aposymbiotic colonies. It is likely that the metabolites produced by the symbiotic holobiont may be more similar in composition between colonies compared to those produced in aposymbiotic colonies, and this difference may help select and enrich for a specific microbiome. Previous work (34) found no differences in the bulk quantities of lipids, proteins, and carbohydrates between symbiotic and aposymbiotic colonies, although it is possible that other previously undetected biochemical differences are related to our observed trends. It is also possible that aposymbiotic colonies may rely more on particle-based feeding during recovery than do symbiotic colonies. Prey capture, and thus the overall holobiont metabolome, could be more variable in aposymbiotic colonies. A prior field-based, seasonal study of *A. poculata* found no differences between the bulk microbiome composition of symbiotic and aposymbiotic colonies (35). Rather, season was found to have a significant influence on the microbiome, indicating that differences in abiotic factors like temperature, light, UV, and nutrients are responsible for seasonal differences in the holobiont. Additionally, prey availability in the seawater likely differs across seasons, but this has not been investigated. By monitoring the microbiome response to disturbance on a high-resolution time scale, our study uniquely allowed us to detect an influence of symbiont state on microbiome response and recovery from a single, laboratory-controlled disturbance. It is important to acknowledge that this study examined changes in the relative and not absolute abundances of microbial community members. Quantitative approaches, including cell counts and quantitative PCR, combined with probe-based imaging of specific taxonomic groups, could help verify the observed trends.

*A. poculata* did not have measurable superoxide at colony surfaces, regardless of symbiotic state or treatment with antibiotics. Concentration of extracellular superoxide associated with corals is documented to be highly species-specific (31) and does not differ with bleaching or density of Symbiodiniaceae (22). Prior measurements by Diaz and colleagues (22) were taken during a natural bleaching event, and our study similarly shows that symbiotic state did not affect extracellular superoxide levels. Similar to *A. poculata*, the stony corals Fungia scutaria, Montipora capitata, Montastraea cavernosa, and Orbicella faveolata also did not have measurable extracellular superoxide at the surface of their colonies (22, 31). In contrast, microbial production of superoxide is widespread (43). In the present study, bacteria previously isolated from *A. poculata* produced extracellular superoxide at rates consistent with those of other heterotrophic bacteria. Both Ruegeria meonggei and Erythrobacter vulgaris demonstrated an inverse relationship between cell number and cell-normalized superoxide production rate, as widely observed in other bacteria, diatoms, and algae (24, 28, 44). The reason for the lack of measurable coral superoxide despite the ability of mucus-hosted bacteria to produce it is unknown, but it may be due to lower production *in situ* or enhanced decay processes by either the microbiome or the coral itself. It is also possible that these bacteria are not producing superoxide while living in the surface mucus layer because cell density is high, although treatment with antibiotics was expected to lower this density. Since superoxide levels did not change upon antibiotic treatment, the coral host may be playing the predominant role in regulating superoxide (via production and/or decay) at the coral surface. In fact, previous research pointed to the primary role of the coral animal in extracellular superoxide at the surface of Porites astreoides colonies (33), yet the underpinning biochemical and environmental controls on superoxide levels remain unknown. It is also possible that growing cultures in isolation artificially inflated per cell production rates, as growth conditions were optimized without competition from other microbes or symbionts. However, in a coculture of *Symbiodinium* strain CCMP2456 and common coral bacterial symbiont Endozoicomonas montiporae strain LMG24815, there was no measurable difference in coculture production rates compared to those in isolated cells (33), suggesting that symbiont interactions may not greatly affect superoxide production, at least under those conditions. Alternatively, coral and/or bacterial superoxide may be rapidly degraded within the mucus via reaction with organic carbon or metals. Clearly, further research is required to understand the controls on, fate of, and basis for extracellular ROS within corals.

As corals experience increasing global and localized threats (18, 19, 45), it is important to understand how the coral holobiont responds. The surface mucus microbiome is critical for coral growth and survival because it acts as the first line of defense against opportunistic pathogens (46–48). Here, we demonstrate that after laboratory manipulation of the mucus microbiome, opportunistic microbes increased in relative abundance. While the microbial communities of both symbiotic and aposymbiotic colonies shifted similarly during recovery, corals harboring symbiont *B. psygmophilum* tended to have bacterial communities with lower beta diversity during this recovery period. This finding suggests a role for symbiotic algae in providing stability and resilience to the microbiome and ultimately the coral holobiont. Both thermal bleaching and disease are major threats to tropical corals, and this work, alongside other work on tropical corals (49, 50), provides a major step toward identifying the microbiome’s roles in maintaining coral resilience, especially the influence of Symbiodiniaceae (absence/presence) on the microbiome composition and processes that drive coral recovery. *A. poculata* is well poised to contribute to this future research due to the overlap in microbial taxa between *A. poculata* and tropical corals (35), its facultative symbiosis with Symbiodiniaceae, and the microbiome disturbance-recovery work presented here. Identifying the intricacies of how the microbiome stabilizes coral health remains a necessary area of future research. Overall, the *A. poculata* symbiotic/aposymbiotic system provides a powerful glimpse into multipartner interactions that contribute to coral holobiont resilience to environmental disturbance.

## MATERIALS AND METHODS

### Coral collections, acclimatization, and pretreatment sampling.

Twenty colonies (10 symbiotic and 10 aposymbiotic) of *Astrangia poculata* were collected from the pier of the Woods Hole Oceanographic Institution (WHOI) from May 30 to July 12, 2017 from 5 to 10 m depth. Colonies (diameter = 25 to 100 mm) were transported to the WHOI Environmental Systems Laboratory (ESL) and held within plastic aquaria where they were exposed to sand-filtered seawater drawn from 4 m depth, 200 m off-shore in Vineyard Sound (flow rate 800 ml min^−1^). Colonies were acclimated 3 to 16 days prior to experimentation. Three days prior to treatment, each colony was gently scrubbed to remove excess sediments and epibionts from the skeletons. Temperature and light data were continuously collected (Table S2) throughout acclimatization and treatment with a HOBO data logger (Onset Data Loggers, Bourne, MA).

Prior to the start of the experiment, five brown (symbiotic) and five white (aposymbiotic) colonies were randomly chosen for the treatment and control groups and placed into two separate 2-liter closed plastic aquaria systems containing 0.22-μm-filter-sterilized seawater. These closed aquaria systems were placed within a larger flowthrough seawater table, allowing colonies within the aquaria to be exposed to ambient seawater temperatures. Pretreatment measurements of extracellular superoxide were then conducted, followed by sampling of coral mucus and surrounding seawater for microbiome analysis. Coral mucus sampling procedures as well as superoxide measurement details are provided in subsequent sections.

### Antibiotic exposure and recovery.

Following pretreatment measurements, the treatment colonies were subjected to 20 ml of antibiotic cocktail (Table 1) mixed into two liters of 0.22 μm filtered seawater. The cocktail was composed of antibiotics that target Gram-positive and Gram-negative bacteria, as well as fungi. Antibiotic selection and exposure time frame (1 week) was based on a previous study of coral mucus (17). The antibiotic treatment and closed aquaria system were maintained for 1 week. Water for each treatment (antibiotic cocktail in filtered seawater for antibiotic treatment and filtered seawater for control treatment) was changed every 12 h.

After treatment, all colonies were placed in new aquaria with 0.22-μm-filtered seawater in the absence of antibiotics for approximately 1 h. Superoxide was measured and mucus and seawater were sampled during this period (0 h time point). Colonies were held within their same antibiotic treatment and control treatment designations and exposed to the sand-filtered seawater (800 ml min^−1^), allowing exposure to 20 μm or smaller size particles. We then followed the recovery of the microbiome by sampling mucus from each colony and seawater from each treatment group at 96 h, 1 week, and 2 weeks.

### Mucus and seawater microbiome collection and processing.

Mucus was collected from each colony by rubbing a sterile cotton swab (HydraFLOCK, Puritan Medical Products, Guilford, ME) over the colony. After the swab was saturated with mucus, it was placed in a cryovial, and the shaft was shortened to approximately 30 mm using scissors sterilized with ethanol. All samples were kept on ice and then transferred to –20°C overnight (for pretreatment swabs) or directly to –80°C (for all other sampling time points) for storage. Ambient seawater (800 or 1,000 ml) was collected from the flow water into the aquaria and filtered through 25-mm diameter, 0.22-μm pore size Durapore membrane filters (Millipore, Boston, MA) using peristaltic pressure filtration and frozen at –80°C. At each time point, water was collected in this manner in duplicate for both the antibiotic and control treatments.

The PowerBiofilm DNA isolation kit (MO BIO Laboratories, Inc., Carlsbad, CA) was used to extract nucleic acids from the *A. poculata* mucus and seawater filters, and the manufacturer’s protocol was followed with the exception of the following steps. Both swabs and filters were placed directly into the bead tube with solutions BF1 and BF2 and mixed briefly with a vortex, and a vortex adaptor was used to bead beat for 15 min after an initial incubation at 65°C. After bead beating was completed, the bead tube was centrifuged and the supernatant was removed to a new sterile tube for each sample. Then, all swabs were inverted so the swab was above the beads and the shaft was at the bottom of the bead tube. The tubes were centrifuged again, the swab was removed, and the remaining supernatant was collected to ensure the maximum amount of material was collected for each sample. The standard protocol was followed thereafter, and nucleic acid yields were assessed using the Qubit dsDNA (double-stranded DNA) high sensitivity fluorescence assay (Invitrogen Corp., Carlsbad, CA) on a Qubit dsDNA fluorometer.

The V4 hypervariable region of the bacterial and archaeal SSU rRNA gene was targeted for amplification through PCR using the barcoded primers 515FY and 806RB (F indicates forward, R indicates reverse, Y and B in both indicate a modification to the original primer set [51, 52]). All reactions were run in triplicate and contained 200 nM each barcoded primer, 1.25 U of GoTaq Flexi DNA polymerase (Promega Corporation, Madison, WI), 5× Colorless GoTaq Flexi Buffer, 200 μM concentrations of each deoxynucleoside triphosphate (dNTP), 2.5 mM MgCl_2_, and <1 to 38 ng of extracted DNA for each sample, for a total reaction mixture volume of 25 μl. Reactions included a positive control, a negative control (sterile water), and a mock community (obtained through BEI Resources, NIAID, and NIH as part of the Human Microbiome Project: Genomic DNA from Microbial Mock Community B [staggered, low concentration, v5.2L, HM-783D]). Although samples from the same time point were extracted at the same time to reduce chances of contamination bias, future studies should include extraction blanks to examine potential sources of contamination (53, 54). Some coral mucus samples were diluted 1:10 to achieve amplification. Reactions were carried out on a thermocycler (Bio-Rad Laboratories, Inc., Hercules, CA) and included an initial denaturation at 95°C for 2 min, followed by 35 to 38 cycles of 95°C for 20 s, 55°C for 15 s, 72°C for 5 min, and a final extension step of 72°C for 10 min. All reaction mixtures were then held at 4°C until screening or gel purification was performed. To ensure amplification, 5 μl of the reaction product was visualized on 1% agarose-TBE gels (Tris-borate-EDTA). To avoid biasing antibiotic or control group samples, 35 cycles was selected as the preferred number of cycles because most mucus samples amplified with these conditions. The HyperLadder II standard (Bioline, Taunton, MA) was used as a reference to detect PCR product that had amplified in the expected region. Two or three PCR replicates were extracted and combined (for a total of 30 μl of product) and purified using the QIAquick gel extraction kit (Qiagen Inc., Valencia, CA, USA). All purified product concentrations were quantified using the Qubit dsDNA HS fluorescence assay so barcoded amplicons could be pooled in equimolar ratios of 5 ng each. This pooled product was sent to the Georgia Genomics and Bioinformatics Core at the University of Georgia for sequencing using 250-bp paired-end MiSeq (Illumina, San Diego, CA) using a previously described approach (55).

### Microbiome data processing and analysis.

All fastq files were processed using mothur version 1.36.1 (56). Sequences that contained ambiguous bases, did not match to the paired end, or were longer than 275 bp were removed, leaving 5,689,940 million sequences which had an average length of 253 bp. The SILVA database (57) was used to align the sequences to the SSU rRNA gene molecule, and chimeric sequences (representing 0.635% of sequences) were removed. The data were classified using the SILVA SSU reference nonredundant (NR) database, release 132 with the k-nearest neighbor algorithm (10 neighbors) in order to remove unknown sequences (190,585 sequences), as well as those from mitochondria (54,361 sequences), eukaryota (1,250 sequences), and chloroplasts (1,503,641 sequences). A distance matrix of the remaining 3,878,910 sequences was created and used to cluster the sequences into OTUs using the average neighbor algorithm at 97% similarity level into 18,483 OTUs. To reduce possible sequencing bias, singleton OTUs (defined as only one occurrence of the OTU in the entire data set) were removed, and this left a data set of 11,169 OTUs, which was used for all subsequent analysis. While the range of sequences present in the data set was large (1,202 to 274,392 sequences), no subsampling was done in order to maintain rare OTUs possibly only present in antibiotic samples, which typically had fewer sequences (58). A total of 8 samples had fewer than 4,000 sequences total, and these were removed from the data set. The negative control, which did not suggest any contamination in the data set, was also removed from the data set during this step.

Statistical analyses were completed using the Primer version 6.1.13 software (PRIMER-E Ltd., Plymouth, United Kingdom) (59) and the open-source programming language R (https://R-project.org/). Relative abundances of the OTUs were square root transformed and compared using Bray-Curtis similarity. Permutational multivariate analysis of variance (PERMANOVA) was performed with Monte Carlo tests to determine if there were significant differences between treatment and time and between symbiotic and aposymbiotic colonies (see Table S1). Similarity percentages (SIMPER) analysis was used on the OTU relative abundance data to identify OTUs that were responsible for 1% or greater dissimilarity between samples, within each treatment group over time. For the 11 OTUs that were found to be significant from this data set, the SINA web aligner (60) and ARB software (61), both with SILVA database version 128, were used to clarify taxonomic identification to genus and species level where possible. The vegan package in R was used to create nMDS plots of beta diversity (62). The Bray-Curtis dissimilarity values were taken for each group from the data used to generate nMDS plots, using the metaMDS vegan function for creation of this matrix. A three-way ANOVA using treatment, time of sampling, and symbiotic state was performed using the R package rstatix. The Sigmaplot package (Systat Software, San Jose, CA) was used for all other statistical analyses.

### Measurement of extracellular superoxide.

Superoxide was measured using a flow injection system (FeLume Mini system, Waterville Analytic, Waterville, ME) as described previously (22). The FeLume system measures chemiluminescence, which in this case results from the mixture of a sample and the superoxide-specific chemiluminescent probe methyl Cypridina luciferin analog (MCLA) (Santa Cruz Biotechnology, Dallas TX) (63). The MCLA reagent contained 50 μM diethylene-triaminepentacetic acid (DTPA) to sequester trace metals, which significantly reduce the lifetime of superoxide. MCLA and the sample to be analyzed were pumped through separate lines at equal rates (2 ml min^−1^) using a peristaltic pump. Mixing occurred in a spiral flow cell, adjacent to the PMT that continuously collected light data, which was transformed to superoxide concentration (see calibration below).

The FeLume was brought to the site of *A. poculata* colonies at the ESL. Background readings were taken in aged filtered seawater and in the surface water of containers with *A. poculata*. To determine superoxide produced by *A. poculata* colonies, steady-state levels were measured in surrounding water at the same depth as colonies but several millimeters horizontally away from colony surfaces. The tube was then moved directly above the surface of the colonies to measure superoxide directly associated with *A. poculata*. The system was flushed with aged filtered seawater or deionized water between control and antibiotic treatment colony sampling.

To measure *in vitro* production of superoxide by *A. poculata* mucus microbes, two bacterial isolates were grown from mucus of *A. poculata* that had been collected at the same WHOI dock where the experimental corals were obtained. Two common coral-associated microbes, *Ruegaria meonggei* and Erythrobacter vulgaris, were selected for analysis. Isolates were grown in duplicate liquid cultures until exponential phase (determined by an optical density of 0.1 to 0.2 at 600 nm). Aliquots of 1 ml were reserved for fixation in 10 μl of 25% glutaraldehyde for cell counting via flow cytometry and for cell loading experiments to determine superoxide production. To perform cell loading, 0.8 ml in 0.2-ml aliquots were loaded onto a 0.22-μm filter inserted into the sample line, directly before the line entered the FeLume as conducted previously (28, 43). Phosphate-buffered saline was run through the sample line and inline filter for 2 min to obtain a baseline, the pump was stopped, cells were gently added to the filter, and flow resumed. Cells were sequentially added upon reaching steady-state signals. At the end of the run, superoxide dismutase (0.8 U ml^−1^) was added to confirm that the cell signal was due to superoxide.

To examine the role of cell number on superoxide production, dilution experiments were performed by diluting mid-exponential-grown cultures 10× and 100× in sterile artificial seawater. After 4 h, superoxide was measured as described above for the cell loading experiments, but 1 ml was initially added to the filter and allowed to reach a steady-state reading.

For calibration, primary standard solutions of potassium dioxide (KO_2_) were prepared in NaOH (pH 12.5) amended with 90 μM DTPA. Superoxide concentrations in primary standards were quantified by measuring the difference in absorbance at 240 nm before and after the addition of superoxide dismutase (SOD; ∼2 U ml^−1^) and then converting to molar units based on the molar absorptivity of superoxide corrected for the absorption of hydrogen peroxide formed during decay at the same wavelength (64). In order to create secondary standards for analysis on the FeLume, these solutions were further diluted in either PBS or TAPS-buffered artificial seawater (481 mM NaCl, 27 mM MgCl_2_·6H_2_O, 10 mM CaCl_2_·2H_2_O, 9 mM KCl, 6 mM NaHCO_3_, 15 mM MgSO_4_·7H_2_O, 3.75 mM tris(hydroxymethyl)methylamino]propanesulfonic acid [pH 8.0], 75 μM DTPA).

For culture measurements, superoxide standards were run with an inline filter without cells to provide consistency with biological experiments and account for any possible artifacts of filtration. The carrier solution was allowed to pass across the filter and react with the MCLA reagent (4.0 μM MCLA, 50 μM DTPA, 0.10 M MES [morpholineethanesulfonic acid] [pH 6.0]) until a stable baseline (<5% coefficient of variation) was achieved for ∼1 min. Then, the secondary standards were pumped directly through the analyte line across the inline filter. Due to the short half-life of superoxide, both primary and secondary standards were prepared immediately before each measurement.

To prepare calibration curves, the chemiluminescence signal generated from the secondary standards was baseline-corrected for chemiluminescence signal arising from the autooxidation of the MCLA reagent. Baseline correction was achieved by subtracting the average background signal generated from the carrier solution passing over the inline filter (without KO_2_) and reacting with the MCLA reagent for at least 1 min. Baseline-corrected chemiluminescence data collected over several minutes of superoxide decay in standard solutions were log-linear and therefore modeled using pseudo-first-order decay kinetics. The half-life of superoxide in most calibrations was typically 2.5 min or less.

Daily calibration curves were generated from three paired observations of time-zero superoxide concentration (dependent variable) and extrapolated chemiluminescence (independent variable) using linear regression. Because chemiluminescence values were baseline-corrected, regression lines were forced through the origin. Calibrations yielded highly linear curves (typically *R*^2^ > 0.9), with a typical sensitivity of 1 chemiluminescence unit per pM superoxide.

### Data availability.

Raw sequences are available at the National Center for Biotechnology Information’s Sequence Read Archive under accession number PRJNA481946. The sequence data are also available via the Coral Microbiome Portal (https://vamps2.mbl.edu/portals/CMP).
