# Supplementary material for: Differential Patterns of Microbiota Recovery in Symbiotic and Aposymbiotic Corals following Antibiotic Disturbance
Source: mSystems. 2021 Apr 13;6(2):e01086-20. doi: 10.1128/mSystems.01086-20 (PMC8546993; doi:10.1128/mSystems.01086-20)
Supplement: TABLE S1 [file msystems.01086-20-st001.pdf]

		PERMANOVA	
		t value	p(MC) value (%)

Treatment

This significance test compared coral community between treatment groups, based on a Bray-Curtis matrix. To mitigate community shifts over time, comparisons were done between the control and treatment group using only colonies from one time point at a time.

		t value	p(MC) value (%)
all coral data			
pre-treatment		0.88269	0.629
0 hours after treatment		2.2465	* 0.001
96 hours after treatment		1.8065	*0.006
one week after treatment		1.6366	*0.007
two weeks after treatment		1.3756	0.061

To determine if the control and treatment groups were receiving seawater that contained similar microbial communities, the extracted product from seawater filters from each time point were compared. To reduce variability due to shifting community structure over the course of the experiment, tests were done to compare only seawater filters collected at the same time point between groups. In most cases, duplicate filters are represented (see Table 1 for exceptions).

		t value	p(MC) value (%)
all seawater data			
seawater (0 hours after)		1.1245	0.349
seawater (96 hours after)		1.0918	0.385
seawater (one week after)		1.2718	0.218
seawater (two weeks after)		1.0308	0.438

Symbiotic State

The effect of the presence or absence of Symbiodium was considered for all colonies before treatment, and then within treatment group at each time point, to separate differences due to changes in colony communities over time and changes due to treatment.

		t value	p(MC) value (%)
pre-treatment, all coral		1.9381	* 0.002
Antibiotic:			
pre-treatment		1.8686	* 0.014
0 hours after treatment		1.4372	0.105
96 hours after treatment		1.8618	* 0.026
one week after treatment		1.0802	0.35
two weeks after treatment		1.1846	0.228
Control:			
pre-treatment		1.2179	0.166
0 hours after treatment		0.85508	0.583
96 hours after treatment		0.99136	0.45
one week after treatment		1.125	0.297
two weeks after treatment		1.144	0.306

Coral vs Seawater

To determine if coral colonies harbored distinctly different communities than the seawater that was flowing over them, the communities extracted from the seawater sample associated with the treatment and time point for each treatment group was compared to the coral colony communities.

		t value	p(MC) value (%)
pre-treatment (all samples)		1.3298	0.067
Antibiotic:			
0 hours after treatment		2.5217	* 0.002
96 hours after treatment	*only 1 SW sample	1.5025	0.062
one week after treatment		2.3743	* 0.002
two weeks after treatment		2.2898	* 0.004
Control:			
0 hours after treatment		1.9332	* 0.014
96 hours after treatment		2.4252	* 0.002
one week after treatment		2.2705	* 0.003
two weeks after treatment		1.9825	* 0.008

Time

It was predicted that the bacterial communities would shift over time, particularly those of the antibiotic treatment group which were exposed to antibiotics and then sampled over a two week recovery period. To determine if the changes between each time point were significant, and how different colonies were from the pre-treatment base, several PERMANOVA tests were done. This was completed for the control group, antibiotic treated, and seawater samples.

		t value	p(MC) value (%)
Antibiotic Coral:	pre-treatment to 0 hours after treatment	2.2055	* 0.002
	pre-treatment to 96 hours after treatment	2.0066	* 0.001
	pre-treatment to one week after treatment	1.9727	* 0.005
	pre-treatment to two weeks after treatment	2.111	* 0.001
	0 hours to 96 hours after treatment	1.6519	* 0.012
	0 hours to one week after treatment	1.8784	* 0.003
	0 hours to two weeks after treatment	2.4035	* 0.001
	96 hours to one week after treatment	1.2078	0.158
	96 hours to two weeks after treatment	1.9946	* 0.002
	one week to two weeks after treatment	1.64	* 0.007
Control Coral:	pre-treatment to 0 hours after treatment	1.3893	0.067
	pre-treatment to 96 hours after treatment	1.5756	* 0.008
	pre-treatment to one week after treatment	1.8191	* 0.003
	pre-treatment to two weeks after treatment	1.7418	* 0.004
	0 hours to 96 hours after treatment	1.2341	0.129
	0 hours to one week after treatment	1.5842	* 0.022
	0 hours to two weeks after treatment	1.6101	* 0.025
	96 hours to one week after treatment	1.2109	0.174
	96 hours to two weeks after treatment	1.3295	0.084
	one week to two weeks after treatment	1.3493	0.069
Seawater (combined control and antibiotic):	pre-treatment to 0 hours after treatment	1.374	0.137
	pre-treatment to 96 hours after treatment	1.6172	0.107
	pre-treatment to one week after treatment	1.3635	0.148
	pre-treatment to two weeks after treatment	1.6309	0.094
	0 hours to 96 hours after treatment	1.5161	* 0.038
	0 hours to one week after treatment	1.7472	* 0.012
	0 hours to two weeks after treatment	2.4907	* 0.003
	96 hours to one week after treatment	1.388	0.091
	96 hours to two weeks after treatment	2.452	* 0.006
	one week to two weeks after treatment	2.2377	* 0.004
