# Supplementary figures and images for: Differential Patterns of Microbiota Recovery in Symbiotic and Aposymbiotic Corals following Antibiotic Disturbance
Source: mSystems. 2021 Apr 13;6(2):e01086-20. doi: 10.1128/mSystems.01086-20 (PMC8546993; doi:10.1128/mSystems.01086-20)

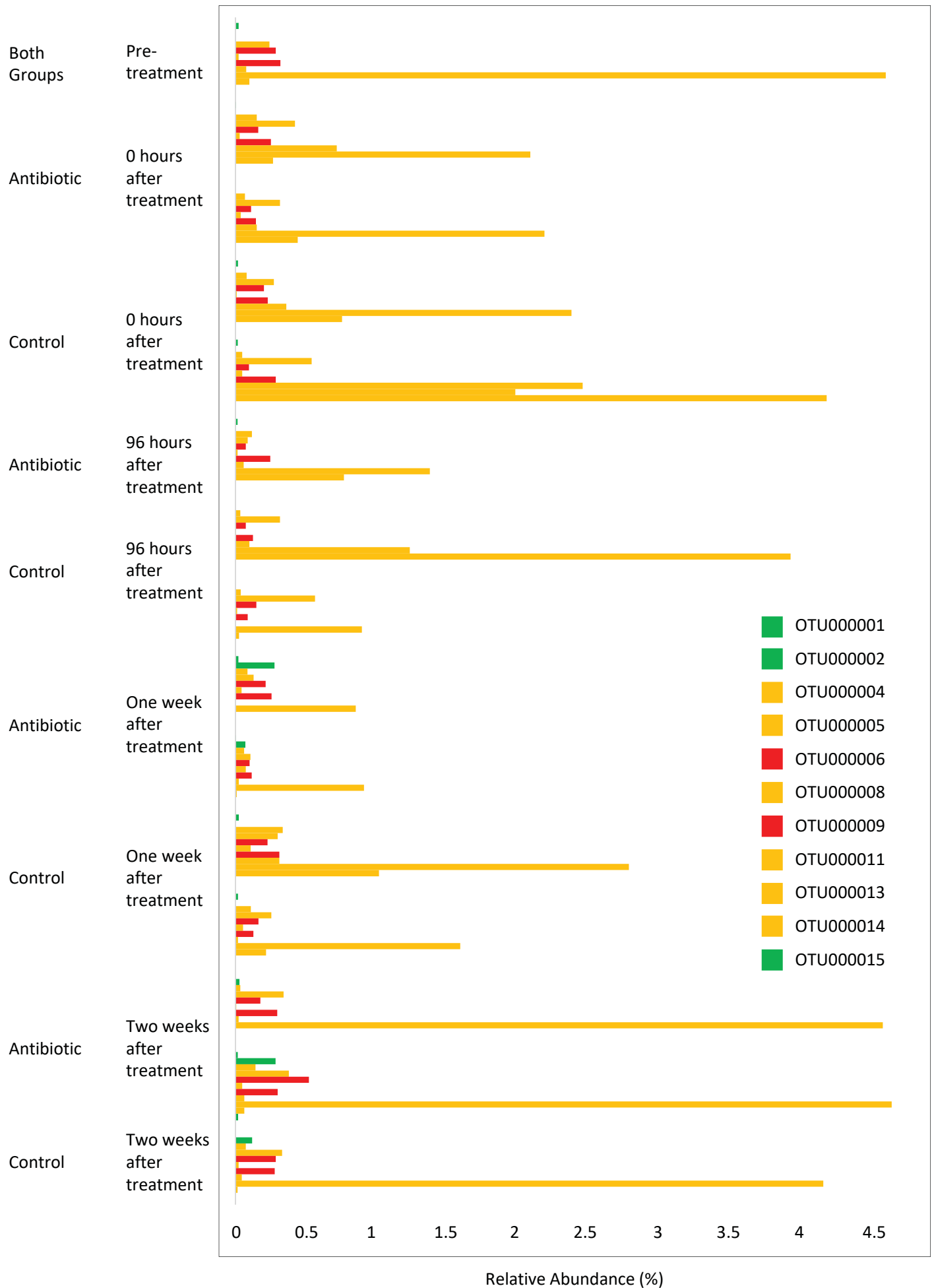

Supplement: FIG S1 [file msystems.01086-20-sf001.pdf]
